# Evaluation of Rice Resistance to Southern Rice Black-Streaked Dwarf Virus and Rice Ragged Stunt Virus through Combined Field Tests, Quantitative Real-Time PCR, and Proteome Analysis

**DOI:** 10.3390/v9020037

**Published:** 2017-02-22

**Authors:** Zhenchao Wang, Lu Yu, Linhong Jin, Wenli Wang, Qi Zhao, Longlu Ran, Xiangyang Li, Zhuo Chen, Rong Guo, Yongtian Wei, Zhongcheng Yang, Enlong Liu, Deyu Hu, Baoan Song

**Affiliations:** 1 State Key Laboratory Breeding Base of Green Pesticide and Agricultural Bioengineering/Key Laboratory of Green Pesticide and Agricultural Bioengineering, Ministry of Education, Guizhou University, Guiyang 550025, China; wzc.4884@163.com (Z.W.); linhong_j@126.com (L.J.); wangwenli0208@163.com (W.W.); fs1989430@gmail.com (Q.Z.); ranlonglu@163.com (L.R.); xiangyangli83@163.com (X.L.); gychenzhuo@aliyun.com (Z.C.); dyhu@gzu.edu.cn (D.H.); 2 College of Pharmacy, Guizhou University, Guiyang 550025, China; 3 College of Life Science, Guizhou University, Guiyang 550025, China; yuji570@163.com; 4 National Agricultural Extension Service Centre, Beijing 100026, China; guorong@agri.gov.cn; 5 Shidian Plant Protection Station, Shidian 678200, China; yongtianwei2016@163.com (Y.W.); sdyzc@163.com (Z.Y.); 6 Mangshi Plant Protection & Quarantine Station, Mangshi 678400, China; enlongliu2016@163.com

**Keywords:** southern rice black-streaked dwarf virus, rice ragged stunt virus, resistance properties, field tests, quantitative real-time polymerase chain reaction, label-free shotgun LC-MS/MS proteomics

## Abstract

Diseases caused by southern rice black-streaked dwarf virus (SRBSDV) and rice ragged stunt virus (RRSV) considerably decrease grain yield. Therefore, determining rice cultivars with high resistance to SRBSDV and RRSV is necessary. In this study, rice cultivars with high resistance to SRBSDV and RRSV were evaluated through field trials in Shidian and Mangshi county, Yunnan province, China. SYBR Green I-based quantitative real-time polymerase chain reaction (qRT-PCR) analysis was used to quantitatively detect virus gene expression levels in different rice varieties. The following parameters were applied to evaluate rice resistance: acre yield (A.Y.), incidence of infected plants (I.I.P.), virus load (V.L.), disease index (D.I.), and insect quantity (I.Q.) per 100 clusters. Zhongzheyou1 (Z1) and Liangyou2186 (L2186) were considered the most suitable varieties with integrated higher A.Y., lower I.I.P., V.L., D.I. and I.Q. features. In order to investigate the mechanism of rice resistance, comparative label-free shotgun liquid chromatography tandem-mass spectrometry (LC-MS/MS) proteomic approaches were applied to comprehensively describe the proteomics of rice varieties’ SRBSDV tolerance. Systemic acquired resistance (SAR)-related proteins in Z1 and L2186 may result in the superior resistance of these varieties compared with Fengyouxiangzhan (FYXZ).

## 1. Introduction

Rice is often subjected to biotic stressors, such as fungi, bacteria, and viruses [1]. In recent years, rice production in China has suffered from two emerging viruses, namely, southern rice black-streaked dwarf virus (SRBSDV) and rice ragged stunt virus (RRSV) [2,3]. SRBSDV is a novel member of the family *Reoviridae* [4,5] and possesses a genome consisting of 10 double-stranded RNA (dsRNA); this virus is prevalent in Southern Asian countries, Vietnam, Japan, and China [6,7,8,9]. The typical symptoms of SRBSDV include dark green and wrinkled leaves, incomplete tassels, tumor-like protrusions ending with small enations, tiller formation on the upper parts of plants, and up-growing rootlets [10,11]. RRSV is a member of the genus *Oryzavirus*. A previous study reported that RRSV contains an icosahedral particle (approximately 65 nm in diameter) with a genome consisting of 10 dsRNA segments [12,13]. RRSV causes diseases with symptoms of twisted leaves, delayed flowering, nodal branch production, incomplete panicle emergence, and panicles bearing mostly unfilled grains; this virus is prevalent in China, Philippines, and Vietnam [14,15,16,17]. The major vectors of SRBSDV and RRSV are white-backed planthopper (WBPH, *Sogatella furcifera*) and brown planthopper (BPH, *Nilaparvata lugens*), respectively [18,19,20]. The epidemic and outbreak of diseases caused by SRBSDV and RRSV are closely associated with the outbreak of viruliferous populations of BPHs and WBPHs [21,22,23,24]. Even at a low density, viruliferous vectors can lead to significant yield loss by virus transmission, especially in China [25]. Although progress has been made in the development of pesticides and controlling technologies against SRBSDV and RRSV, resistant rice cultivars still must be determined to ensure safe production and to identify their resistance characteristics.

This study aimed to evaluate rice cultivars with high resistance to virus. Field trials were conducted to compare factors affecting virus resistance sources. Acre yield (A.Y.) and incidence of infected plants (I.I.P.) were first determined in 22 different rice varieties in Jiucheng Village, Yunnan province of China, in the year of 2012. A.Y. means yield of each rice cultivar per acre. I.I.P. was defined as the mean density of the diseased plants. Viral quantification was an essential tool for the study of resistance mechanisms of plants [26]. Thus, SYBR Green I-based quantitative real-time polymerase chain reaction (qRT-PCR) analysis was then applied to quantitatively detect SRBSDV and RRSV gene expression levels in these varieties. Virus load (V.L.) was determined by comparing the relative mRNA expression levels of the entire SRBSDV and RRSV viral genome through the qRT-PCR method. The resistance of 10 major rice cultivars to SRBSDV and RRSV was simultaneously tested in Jiucheng and Zhefang Village by using an evaluating-parameter, such as the disease index (D.I.) and insect quality (I.Q.), in Yunnan province of China, in the following year of 2013. I.I.P., A.Y., V.L., D.I., and insect I.Q. were applied to evaluate the resistance of all the rice cultivars. Results showed that Zhongzheyou1 (Z1) and Liangyou2186 (L2186) were the most suitable varieties. The underlying resistance mechanism of Z1 and L2186 was investigated, with a common susceptible rice variety as comparison, namely, Fengyouxiangzhan (FYXZ). Comparative proteomic approaches were conducted to comprehensively describe the proteomics of the SRBSDV tolerance of Z1, L2186, and FYXZ. Quantitative label-free liquid chromatography tandem-mass spectrometry (LC-MS/MS) proteomic technology identified systemic resistance-related proteins in SRBSDV-infected cultivars. This study provides the first description of rice resistance properties through field trials, qRT-PCR, and label-free shotgun LC-MS/MS proteomics. Z1 and L2186 were the most suitable varieties with the lowest disease index, insect quantities, and virus accumulation, as well optimal induced systemic resistance.

## 2. Materials and Methods

### 2.1. Field Tests

All tested rice cultivars were sown in a one-row plot with three replicates in the experiment field (Jiucheng Village, Shidian County, Yunnan Province, China) during a period of SRBSDV and RRSV epidemic in the prefecture in 2012. The plants were transplanted to the field in early July and harvested in mid-November. I.I.P. and A.Y. of these rice varieties were determined in 2012. Twenty-two listed rice varieties were investigated (Table 1); these varieties include Neixiangyou18 (N18), Shenzhou5 (S5), L2186, Chuanyou3727 (C3727), IIYou6 (IIY6), Liangyou15 (L15), IIYou629 (IIY629), Mengyou19 (M19), Deyou8 (D8), Yingxiang1 (Y1), YLiangyou696 (Y696), Yunguang17 (Y17), Chuannong2You498 (C2Y498), Huxiang658 (H658), Liangyou816 (L816), Neixiangyou1 (N1), Kefeng182 (K182), Liangyou2161 (L2161), Z1, Nei5You39 (N5Y39), Yingxuan1 (YX1), and IIYou58 (IIY58). All of the cultivars were indica hybrid rice type and most were cultivated in China. Meanwhile, 22 rice cultivars were not transgenic.

Ten rice cultivars from these 22 rice varieties were separately evaluated in Jiucheng and Zhefang Village (Mangshi City, Yunnan Province, China) in 2013. These varieties were uniformly sown on 4 June in Jiucheng Village and 17 April in Zhefang Village, according to the actual production seeding rate. All the plots in 2013 were cultivated in open field with three repetitions and arranged in a randomized block design. I.Q. per 100 clusters was surveyed every week. Three points were investigated in each plot by the diagonal sampling method, and 10 clusters were surveyed in each point during the maturity stage of rice. Each tiller of a cluster was classified according to the disease index classified standard of SRBSDV. The disease index standard was divided into five levels, including level 0 (healthy asymptomatic tillers with normal height), level 1 (tillers with no obvious dwarf symptom, yet had tumor-like protrusions ending with small enations), level 2 (three-quarters of the normal plant height), level 3 (one-half of the normal plant height), and level 4 (one-third of the normal plant height). Five points were investigated in each plot by the cross-sampling method, and five clusters were surveyed in each point. The number of diseased tillers in different levels was investigated to calculate the disease index. The same investigation and classification were used in the two villages.

D.I. = Σ [(total number of diseased tillers in different levels × actual level)/(total number of investigated tillers × the highest level)] × 100(1)

### 2.2. Quantification of SRBSDV and RRSV Gene Expression Levels by qRT-PCR

qPCR primers were designed according to the conserved regions of SRBSDV and RRSV identified within the total sequence to develop a real-time PCR detection system (Tables S1 and S2). All primers used for RRSV amplification were designed by Primer 5.0 software. The primers used for SRBSDV amplification were designed by Beacon Designer 7.7 and synthesized as previously described [27]. Total RNA was extracted from 100 mg of infected rice tissues by using RNAiso Plus (TaKaRa, Dalian, Liaoning, China) according to the manufacturer’s instructions. First-strand cDNA templates were synthesized using 2 μg of total RNA from various samples by using Moloney murine leukemia virus (M-MLV) reverse transcriptase (TaKaRa) with a random primer as the anchor primer. After the components were mixed, the sample was incubated at 42 °C for 1 h and then heated at 70 °C for 15 min. The obtained cDNA was used as the qPCR template. SYBR^®^ *Premix Ex Taq*^TM^ (TaKaRa) was used in all qPCR tests.

Before qRT-PCR, normal RT-PCR was performed using rTaq DNA polymerase (TaKaRa) with each primer pair to ensure that correct products were amplified and that no primer dimer was present. The amplified products were detected by 2% (*w*/*v*) electrophoresis agarose gel. Normal RT-PCR reactions for each sample were performed at 72 °C for 10 min, followed by denaturation at 94 °C for 4 min and 35 amplification cycles of 94 °C for 30 s, 58 °C for 30 s, and 72 °C for 30 s. The non-specific amplification of SRBSDV-cDNA and RRSV-cDNA was significantly inhibited in this assay, and all RRSV products were sequenced. Real-time RT-PCR methods were performed using a SYBR *Premix Ex Taq*^TM^ and iCycle iQ (Bio-Rad, Hercules, CA, USA) detection system with viral transcripts as the template to quantitatively detect SRBSDV and RRSV in rice tissues. The mixture (20 μL) contained 2× SYBR *Premix Ex Taq*^TM^ buffer, 10 μM each of forward primer, reverse primer, and template cDNA, and ice-cold sterilized water. The reaction mixtures were incubated at 95 °C for 30 s, followed by 40 cycles of 95 °C for 5 s and 60 °C for 30 s. The mRNA level was quantified in relation to the expression of rice 18S *rRNA* (GenBank Acc. No. AK059783).

Twenty-two rice varieties under the same treatment with three parallel repetitions at the dough stage were used for qRT-PCR analysis. Suspected singly infected or co-infected SRBSDV and RRSV rice specimens with typical dwarf symptoms were obtained from diseased fields and stored in the laboratory at −80 °C. After the candidates were confirmed to be infected with SRBSDV or RRSV by normal RT-PCR detection, the enations on rice plants were separated by RNA isolation for qRT-PCR analysis. Negative control samples were obtained from rice plants grown in a greenhouse.

### 2.3. Quantitative Label-Free Shotgun Proteomic Analysis of Three Rice Varieties

Recently, by using a comparative proteomic analysis approach, a set of differentially expressed proteins has been successfully identified and characterized to study the difference mechanisms of tolerant and sensitive species [28,29]. In the present study, in order to investigate the resistance characteristics of Z1 and L2186 compared to susceptible control FYXZ, SRBSDV infestation of these three varieties was performed in Jiucheng village during the SRBSDV epidemic period in 2014. The stem tissues from single SRBSDV infestation and healthy clusters were collected and maintained at −80 °C for further use. Total protein from the rice plants was extracted by a modified trichloroacetic acid (TCA)/acetone procedure [30]. About 1500 mg of fresh weight stem powder was suspended in 10 mL of 0.015 g of polyvinylpyrrolidone (PVPP, Sigma, Beijing, China), 10% trichloroacetic acid (TCA, Sigma) in acetone, and 0.07% beta-mercaptoethanol (Sigma). The mixture was incubated at −20 °C overnight, and the extract was centrifuged at 8000 × *g* and 4 °C for 15 min. The pellet was collected and washed with ice-cold acetone by centrifugation at 8000 × *g* and 4 °C for 15 min. The acetone washing step was repeated three times. The colorless resulting pellet was lyophilized in a vacuum centrifuge, and protein quantification was performed by Bradford assay with bovine serum albumin (BSA, Sigma) as the standard [31].

The extracted proteins in sodium dodecyl sulfate (SDS) sample buffer were separated on 10% SDS-polyacrylamide gel electrophoresis (PAGE). After electrophoresis, the gels were categorized into four groups according to the standard protein molecular weight, before protein visualization with colloidal Coomassie G-250 overnight. The samples were air dried and reduced with 10 mM Dithiothreitol (DTT, Solarbio Co. Ltd, Sigma)/50 mM NH_4_HCO_3_ (pH 8.0, Sigma) at 56 °C for 1 h before alkylation in the dark for 30 min with 55 mM iodoacetamide (Sigma-Aldrich, Beijing, China)/50 mM NH_4_HCO_3_ (pH 8.0). The samples were washed with 10 mM NH_4_HCO_3_, and 100% acetonitrile (ACN, Sigma) twice for 10 min each and then air dried. Finally, samples were digested with trypsin in 10 mM NH_4_HCO_3_ at 37 °C for 8 h or overnight. Peptides resulting from trypsin digestion of proteins were extracted with equal volumes of 60% ACN/5% formic acid (FA, Sigma) solution, dried, vacuum centrifuged, and reconstituted in 50 μL of H_2_O (HPLC grade, Wahaha, Hangzhou, Zhejiang, China) containing 0.1% FA for LC-MS/MS analysis.

The tryptic digest extracts from one-dimensional electrophoresis (1 DE) gel slices were analyzed by a LC-MS/MS system according to a previous study [32]. Samples were automatically injected onto the C18 reversed-phase column (3C18-CL, 75 μm × 15 cm, CA). A 2.3 kV electrospray voltage was applied via a liquid junction upstream of the C18 column. Each sample was loaded onto the C18 column and subjected to an initial step in gradient elution mode at a flow rate of 300 nL/min, with 95% solvent B (95% ACN, 5% HPLC grade water + 0.1% *v*/*v* FA) and 95% solvent A (5% ACN, 95% HPLC grade water + 0.1% *v*/*v* FA). The 5600 Triple time-of-flight (TOF) MS was operated in data-dependent mode and automatically switched between TOF-MS and product ion acquisition in Analyst (R) Software (TF1.6).

MaxQuant 15 version 1.5.2.8 was used to analyze and quantify the file of “wiff”. The protein database of *Oryza sativa* and southern rice black-streaked dwarf virus from UniProt was used to search the MS/MS spectra by the Andromeda search engine [33]. For peptide and protein identifications, the false discovery rate (FDR) was set to 1.0%. For label-free quantification, the iBAQ algorithm was used to rank the absolute abundance of different proteins within a single sample [34], and iBAQ data were used for the Student’s *t*-test. The differentially expressed proteins were filtered by the following cutoff: *p*-value was lower than 0.05 for the *t*-test. To evaluate the false positive rate of this approach, we constructed a reversed sequence databank (a database in which the sequences have been reversed) containing the same number of proteins in the *Oryza sativa* and southern rice black-streaked dwarf virus database.

## 3. Results

### 3.1. Field Yields Test of 22 Rice Candidates in Jiucheng in 2012

**Figure 1:**
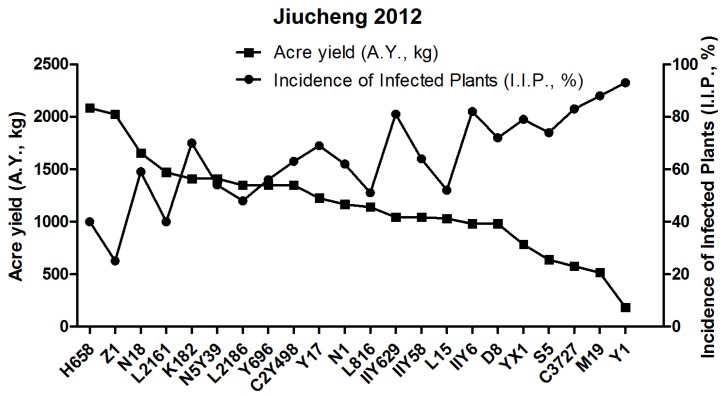
Correlation between incidence of infected plants (I.I.P., %) and acre yield (A.Y., kg) of 22 rice varieties in the 2012 field test.

In early July of 2012, rice varieties were cultivated in Jiucheng during the explosion of SRBSDV and RRSV. The I.I.P. and A.Y. of 22 candidates were investigated. The yield test showed that 22 candidates were categorized into four classes, in which H658 (A.Y., 2085.05 kg) and Z1 (A.Y., 2023.74 kg) with the highest harvest yields included in Group 1, and YX1, S5, C3727, M19, Y1 with the lowest harvest yields were named as Group 4, the A.Y. of which range from 183.92 to 784.85 kg. Among those remaining, C2Y498, Y696, L2186, N5Y39, K182, L2161, N18 belonged to Group 2 and had higher harvest yields ranging from 1348.75 to 1655.29 kg. Group 3 consisted of eight varieties including D8, IIY6, L15, IIY58, IIY629, L816, N1, Y17 with lower harvest yields ranging from 980.91 to 1226.14 kg (Figure 1 and Table 1).

### 3.2. Analysis of the Relative mRNA Expression Level of SRBSDV and RRSV Genes

The relative mRNA expression quantity of all SRBSDV and RRSV genes from various infected rice varieties was monitored through the qRT-PCR method to determine its relation to the resistance of the rice varieties. Normal RT-PCR was performed using cDNA derived from samples co-infected with SRBSDV and RRSV to verify the specificity of the primers used in the study. Electrophoresis analysis showed that 13 pairs of primers based on SRBSDV (Figure S1 and Table S1) and 12 pairs of primers based on RRSV (Figure S2 and Table S2) produced stable and specific bands, respectively. After sequencing, the result was tested using BLAST and compared with the corresponding subtypes of SRBSDV and RRSV. Melting curve analysis showed single peaks for all samples tested. The results proved that the primers were suitable for qPCR tests.

**Figure 2:**
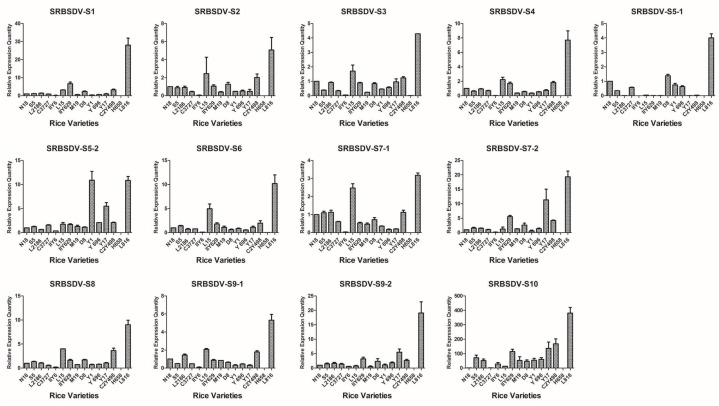
Map generated from the responses of 15 dual infestation rice varieties to 13 SRBSDV genomes. All rice varieties consisted of N18 (1), S5 (2), L2186 (3), C3727 (4), IIY6 (5), L15 (6), IIY629 (7), M19 (8), D8 (9), Y1 (10), Y696 (11), Y17 (12), C2Y498 (13), H658 (14), and L816 (15).

**Figure 3:**
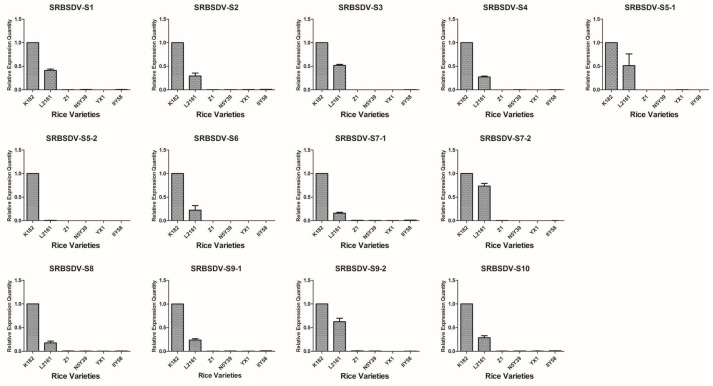
Map generated from the responses of six single infestation rice varieties to 13 SRBSDV genomes. All rice varieties consisted of K182, L2161, Z1, N5Y39, YX1, and IIY58.

**Figure 4:**
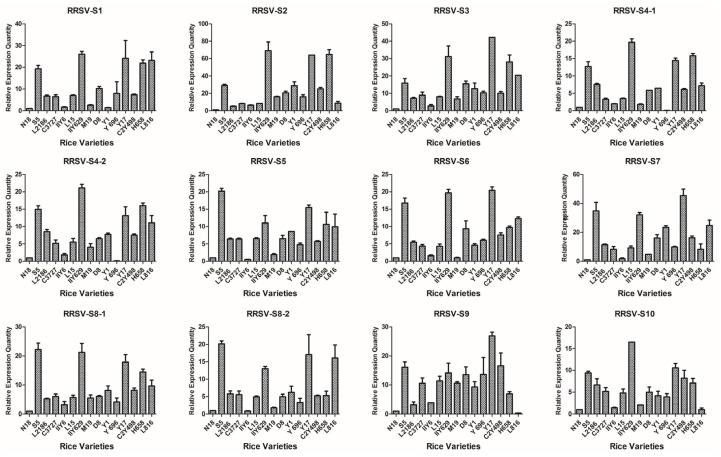
Map generated from the responses of 15 dual infestation rice varieties to 12 RRSV genomes. All rice varieties consisted of N18 (1), S5 (2), L2186 (3), Chuanyou3727 (4), IIY6 (5), L15 (6), IIY629 (7), M19 (8), D8 (9), Y1 (10), Y696 (11), Y17 (12), C2Y498 (13), H658 (14), and L816 (15).

Based on the detection results of candidate varieties in 2012, 15 rice varieties were confirmed to be co-infected with SRBSDV and RRSV; one variety was singly infected with RRSV, and six varieties were singly infected with SRBSDV; at least triplicate candidates were used (Table 1). Virus load in all the rice tissues was correlated with the severity of infection and thus may be used as an indicator for evaluating the infection susceptibility of different cultivars. The V.L. of 22 rice varieties were evaluated using the virus content measured according to the 2^−ΔΔC*t*^ algorithm with the defining values of the SRBSDV genomes of N18, RRSV genomes of N18, and SRBSDV genomes of K182 as 1.00 (Figure 2, Figure 3 and Figure 4).

The V.L. of 15 dual infestation rice varieties in relation to SRBSDV exhibited a particular trend (Figure 2). L816 showed the highest V.L., whereas H658 and IIY6 showed the lowest. For instance, the expression levels of SRBSDV-S1 in L816, H658, and IIY6 were 28.04-, 0.005-, and 0.17-fold higher, respectively, than that of N18. Overall, S5, L2186, C3727, L15, IIY629, M19, D8, Y1, Y696, Y17, C2Y498, and N18 had no evident variation tendency, with the following exceptions: Y1 and Y17 had 10.90- and 5.48-fold higher V.L. than that of N18 of the S5-2 gene, and L15 had 4.97- and 4-fold higher V.L. than that of N18 of S6 and S8 genes. The V.L. to SRBSDV in single infestation was also determined. The entire contents of the SRBSDV genome decreased greatly in Z1, N5Y39, YX1, and IIY58 compared with that in K182 and L2161 (Figure 3). Briefly, H658, IIY6, Z1, N5Y39, YX1, and IIY58 had lower V.L. to SRBSDV than other cultivars.

The V.L. of 15 dual infestation rice varieties to RRSV significantly differed from that to SRBSDV (Figure 4). For example, RRSV content was higher in S5, IIY629, Y17, and H658 than that in the other cultivars. The corresponding V.L. was also slightly higher in D8 and C2Y498. The RRSV levels in N18 and IIY6 were the lowest among all the rice varieties. The RRSV levels in L2186, C3727, and L15 were slightly lower than those in the other varieties. The contents of RRSV in the remaining rice varieties such as M19, Y1, Y696, C2Y498, and L816 were detected at the mid-level. Hence, N18, IIY6, L2186, C3727, and L15 exhibited lower V.L. to RRSV than that of the other varieties.

### 3.3. Field Yields Tests of 10 Major Rice Cultivars in Jiucheng and Zhefang in 2013

**Figure 5:**
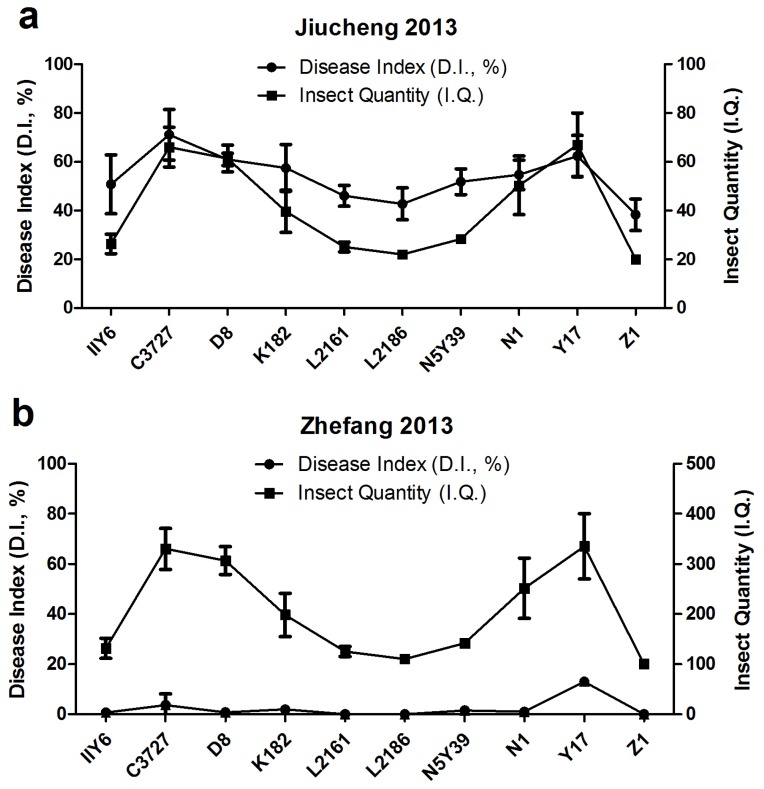
Field tests in 2013. (**a**) Relationship between the disease index (D.I.) and insect quantity per 100 clusters (I.Q.) of 10 rice cultivars in Jiucheng village. (**b**) Relationship between the disease index (D.I.) and insect quantity per 100 clusters (I.Q.) of 10 rice cultivars in Zhefang village.

Twenty-two rice varieties were subjected to preliminary assessments on I.I.P., A.Y., and V.L. to SRBSDV and RRSV, which was obtained from the field test in the first year. Ten rice cultivars among the four groups were screened to re-evaluate the resistance to the virus in the following year. A comparison of the field test results using 10 rice cultivars from Jiucheng showed that breeding cultivars Z1, L2161, and L2186 performed better than other varieties; these three cultivars produced the lowest D.I. and I.Q. values (Figure 5a). In Zhefang, despite the absence of evident variation in I.Q., the cultivars Z1, L2161, and L2186 exhibited the highest resistance level, as measured by D.I., with the lowest values compared with others (Figure 5b).

### 3.4. Proteomic Analysis in Three Healthy and SRBSDV-Stressed Rice Cultivars

A comparative stem proteomic analysis of rice under healthy and infected conditions with SRBSDV was performed to investigate the resistance characteristics of Z1 and L2186, with the susceptible variety FYXZ as the contrast. Proteins from a healthy control and SRBSDV-infected stem samples of three rice varieties were extracted and analyzed using a quantitative label-free proteomics approach. The list of all identified and quantified proteins in cultivars was presented in Tables S3 and S4.

#### 3.4.1. Overview of the Proteomic Analysis of Three Healthy Cultivars

**Figure 6:**
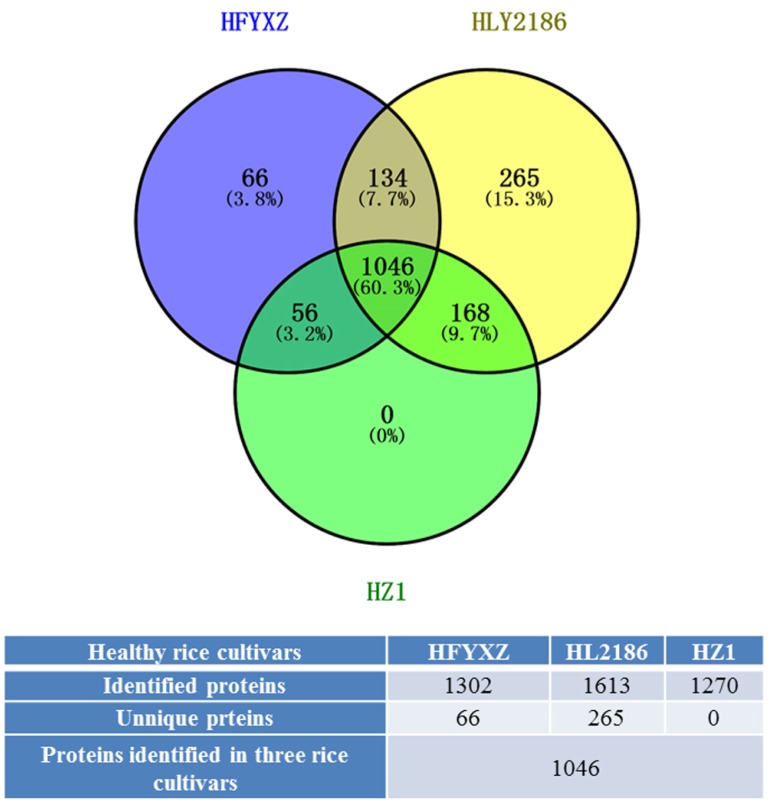
Distribution and overlap of rice proteins identified in healthy rice varieties. Blue circle: healthy FYXZ cultivars (HFYXZ); Yellow circle: healthy L2186 (HL2186); Green circle: healthy Z1 (HZ1).

A total of 1302 proteins were identified in healthy FYXZ cultivars (HFYXZ), and 1613 in healthy L2186 (HL2186), and 1270 in healthy Z1 (HZ1). The majority of the 1046 proteins (60.3%) were detected under three varieties, while 66 (3.8%) were uniquely identified in HFYXZ, and 265 (15.3%) in HL2186. A total of 320 up-regulated and 186 down-regulated proteins in the HL2186 vs. HFYXZ group were differentially expressed (flod changes > 1.5, *p* < 0.05); 301 up-regulated and 300 down-regulated proteins in the HZ1 vs. HFYXZ group were differentially expressed (flod changes > 1.5, *p* < 0.05); 220 up-regulated and 303 down-regulated proteins in the HZ1 vs. Hl2186 group were differentially expressed (flod changes > 1.5, *p* < 0.05) (Figure 6 and Tables S3 and S4).

#### 3.4.2. Overview of the Proteomic Analysis in Three SRBSDV-Stressed Cultivars

**Figure 7:**
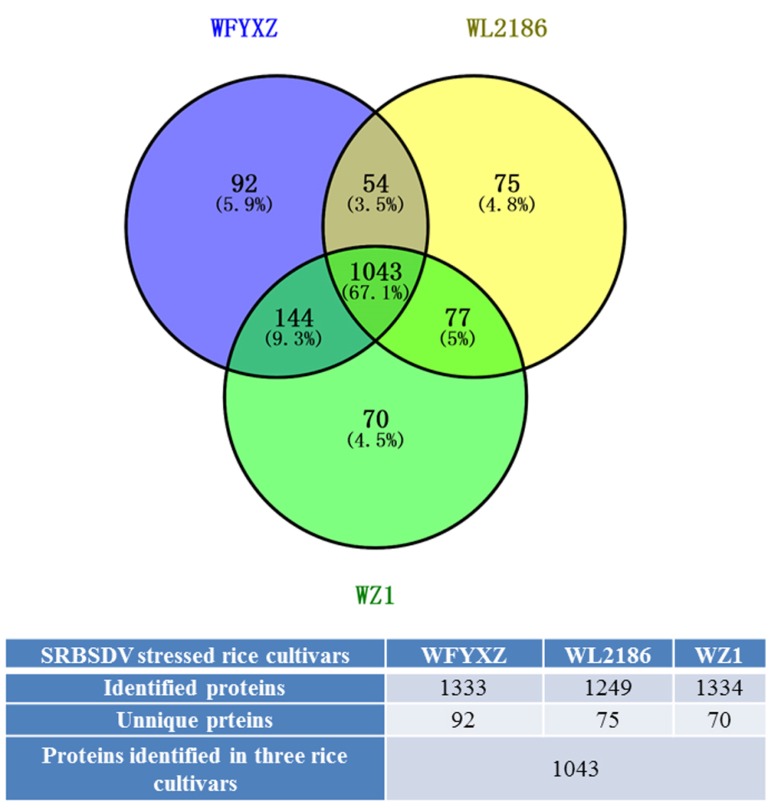
Distribution and overlap of rice proteins identified in SRBSDV-stressed varieties. Blue circle: SRBSDV-stressed FYXZ cultivars (WFYXZ); Yellow circle: SRBSDV-stressed L2186 (WL2186); Green circle: SRBSDV-stressed Z1 (WZ1).

A total of 1333 proteins were identified in SRBSDV-stressed FYXZ cultivars (WFYXZ), 1249 in SRBSDV-stressed L2186 (WL2186), and 1334 in SRBSDV-stressed Z1 (WZ1). The majority of the 1043 proteins (60.3%) were detected under three varieties, while 92 (5.9%) were uniquely identified in WFYXZ, 75 (4.8%) in WL2186, and 70 (4.5%) in WZ1. A total of 255 up-regulated and 383 down-regulated proteins in the WL2186 vs. WFYXZ group were differentially expressed (flod changes > 1.5, *p* < 0.05); 235 up-regulated and 220 down-regulated proteins in the WZ1 vs. WFYXZ group were differentially expressed (flod changes > 1.5, *p* < 0.05); 342 up-regulated and 190 down-regulated proteins in the WZ1 vs. Wl2186 group were differentially expressed (flod changes > 1.5, *p* < 0.05) (Figure 7 and Tables S3 and S4).

#### 3.4.3. Systemic Resistance-Related Proteins

In HL2186, three proteins associated with systemic acquired resistance (SAR): dolichyl-diphosphooligosaccharide-protein glycosyltransferase, hypoxia up-regulated protein, and membrane-attack complex (MAC); they were up-accumulated than HFYXZ. In HZ1, four proteins associated with SAR: dolichyl-diphosphooligosaccharide-protein glycosyltransferase, hypoxia up-regulated protein, fatty acyl-CoA synthetase, and hydroxyproline-rich glycoprotein-like; they were up-accumulated than HFYXZ. In WL2186, three proteins associated with SAR: mitogen-activated protein kinase, ATP synthase B chain protein, and sedoheptulose-1,7-bisphosphatase; they were up-accumulated than WFYXZ. In WZ1, four proteins associated with SAR: mitogen-activated protein kinase, sedoheptulose-1,7-bisphosphatase, dolichyl-diphosphooligosaccharide-protein glycosyltransferase, and MAC; they were up-accumulated than WFYXZ. In addition, pathogenesis-related protein 1 (PR1) and pathogenesis-related protein 10 (PR10) were substantially up-regulated in WL2186; pathogenesis-related protein 3 (PR3) was substantially up-regulated in WZ1 (Tables S3 and S4).

## 4. Discussion

Investigation into the relationship between rice varieties and anti-SRBSDV or anti-RRSV features is important. Considering that the virus genome expression levels are relative to those of susceptible varieties [35], scholars have established a new parameter, namely, V.L. The intensity of V.L. can be measured using the genome variation tendency response in 22 rice varieties. The lowest SRBSDV quantities of H658 and IIY6 showed excellent anti-virus features to SRBSDV, and L816 exhibited almost no anti-virus features to SRBSDV compared with that of other varieties. Moreover, Z1, N5Y39, Y1, and IIY58 exhibited higher anti-virus features to SRBSDV than K182 and L2161. RRSV quantity data revealed that N18 and IIY6, as well as L2186, C3727, and L15, exhibited anti-virus features to RRSV, and the former group was superior to the latter group. However, six varieties, including S5, IIY629, Y17, H658, D8, and C2Y498, were more susceptible to RRSV. In conclusion, singly infected rice varieties, such as Z1, N5Y39, K182, L2161, and N1, and three varieties, namely, L2186, C3727, and IIY6, with higher anti-virus features were reevaluated in the following year. Meanwhile, two susceptible varieties, namely, Y17 and D8, to RRSV were also selected in our following year’s field trials.

Based on the field test in the first year, a series of field tests was performed in separate districts to evaluate the resistance of Z1 from Group 1; C3727 from Group 4; L2161, K182, N5Y39, and L2186 from Group 2; and Y17, N1, IIY6, and D8 from Group 3. Z1, L2161, and L2186 were superior to other varieties, as indicated by their high A.Y., low I.I.P. and D.I., and improved anti-virus features and I.Q. performance. However, C3727 and Y17 still exhibited the highest D.I. and I.Q. performance. The two years’ field tests results of IIY6 indicated that this variety was not suitable for cultivation despite its lower V.L. of SRBSDV and RRSV. The remaining four rice cultivars such as K182, N1, N5Y39, and D8 could not be cultivated. Among the tested rice cultivars, Z1 and L2186 were deduced to be the most suitable varieties because of their low I.I.P., D.I., I.Q., V.L. and high A.Y. features.

A comparative stem proteomic analysis of rice under healthy and infected conditions with SRBSDV was performed to investigate the resistance characteristics of Z1 and L2186, with the susceptible variety FYXZ as the contrast. Hierarchical clustering was conducted to visualize the coordinately regulated proteins. Two major clusters were produced; each of them consisted of three healthy and SRBSDV-infected rice samples. Z1 and L2186 were further classified to the same sub-cluster, which differed from FYXZ (Figure S3). Plants had induced resistance, which included partial resistance (hypersensitive response, HR) and systemic resistance (SAR) and occurred as a result of virus infection [36]. Due to no differential protein being involved in HR, we focused on SAR-related proteins (Table S4). SAR is the plant’s immune response to pathogen attack and is a component of plants’ integrated disease-resistance repertoire [37,38,39,40]. In the present study, among the SAR-related proteins, hypoxia up-regulated protein, mitogen-activated protein kinase, and ATP synthase B chain protein had ATP binding activity. Intriguingly, dolichyl-diphosphooligosaccharide-protein glycosyltransferase plays important roles in the regulation of defense responses in plants [41]. In response to pathogen infection, a sequential and highly specific interaction between the constituent elements occurs to form transmembrane channels which are known as the membrane-attack complex (MAC). The MAC provides plant immunity [42,43]. Furthermore, pathogenesis-related proteins showed substantial up-regulation features in WL2186 and WZ1. It is generally thought that SAR results from the concerted effects of many PR proteins [39]. The PR-protein family is a common class of water-soluable proteins generated by plants in response to pathogenic infection and stimulation by abiotic factors. PR-1 is a glycine-rich disease resistance protein; PR-3 has β-1,3-glucanase and chitinase activities, respectively [44,45]. These proteins were up-regulated in WL2186 and WZ1 infected with SRBSDV, suggesting that it may activate SAR to resist SRBSDV. Different behaviors of SAR-related proteins in Z1 and L2186 may result in the superior resistance of these varieties compared with FYXZ.

## 5. Conclusions

The most economical and effective way of controlling SRBSDV and RRSV diseases is the use of culture-resistant rice varieties. However, a comprehensive resistant variety has not yet been discovered. In this paper, the prospects of developing rice cultivars with durable resistance to these viruses were discussed by analyzing three parameters—I.I.P., A.Y., and V.L.—in an experiment. The resistance tests of 10 major rice cultivars were conducted by measuring D.I. and I.Q. Finally, this work represents the most extensive proteomic description of SRBSDV responses of Z1 and L2186 and contributes knowledge on virus tolerance in rice varieties. To sum up, Z1 and L2186 exhibiting SAR were found to be the most suitable varieties in the field among all the tested rice cultivars.

## Figures and Tables

**Table 1:** 22 Rice varieties tested in Jiucheng in 2012.

Group Number	Abbreviation	Name of Variety	Infected with SRBSDV (+/−)	Infected with RRSV (+/−)
Group 1	H658	Huxiang658	+	+
	Z1	Zhongzheyou1	+	−
Group 2	N18	Neixiangyou18	+	+
	L2161	Liangyou2161	+	−
	K182	Kefeng182	+	−
	N5Y39	Nei5You39	+	−
	L2186	Liangyou2186	+	+
	Y696	YLiangyou696	+	+
	C2Y498	Chuannong2 You 498	+	+
Group 3	Y17	Yunguang17	+	+
	N1	Neixiangyou1	−	+
	L816	Liangyou816	+	+
	IIY629	IIYou629	+	+
	IIY58	IIYou58	+	−
	L15	Liangyou15	+	+
	IIY6	IIYou6	+	+
	D8	Deyou8	+	+
Group 4	YX1	Yingxuan1	+	−
	S5	Shenzhou5	+	+
	C3727	Chuanyou3727	+	+
	M19	Mengyou19	+	+
	Y1	Yingxiang1	+	+
Control	FYXZ	Fengyouxiangzhan	+	−

SRBSDV: southern rice black-streaked dwarf virus; RRSV: rice ragged stunt virus.

## Supplementary Materials

The following are available online at www.mdpi.com/1999-4915/9/2/37/s1, Figure S1: Detection for specificity of primers designed for SRBSDV by conventional PCR, Figure S2: Detection for specificity of primers designed for RRSV by conventional PCR, Figure S3: Hierarchical clustering analysis of differentially expressed proteins in Z1, L2186, and FYXZ. Table S1: Primers used for the expression level analysis of SRBSDV, Table S2: Primers used for expression level analysis of RRSV, Table S3: List of identified proteins, Table S4: The differential expression proteins.

## References

[1] Yan J.K., Shiping W. (2012). Toward an understanding of the molecular basis of quantitative disease resistance in rice. J. Biotechnol..

[2] Wang Q., Yang J., Zhou G.H., Zhang H.M., Chen J.P., Adams M.J. (2010). The complete genome sequence of two isolates of southern rice black-streaked dwarf virus, a new member of the genus *Fijivirus*. J. Phytopathol..

[3] Jiao D.S., Guo N.M., Chen H.Y., Akita F., Xie L.H., Omura T., Wei T.Y. (2012). Assembly of the viroplasm by viral non-structural proteins Pns 10 is essential for persistent infection of rice ragged stunt virus in its insect vector. J. Gen. Virol..

[4] Zhou G.H., Wen J.J., Cai D.J. (2008). Southern rice black-streaked dwarf virus: A new proposed *Fijivirus* species in the family *Reoviridae*. Chin. Sci. Bull..

[5] Zhang H.M., Yang J., Chen J.P. (2008). A black-streaked dwarf disease on rice in China is caused by a novel *Fijivirus*. Arch. Virol..

[6] Cuong H.V., Nguyen V.H., Vu T.M., Matsumoto M. (2009). Rice dwarf disease in North Vietnam in 2009 is caused by southern rice black-streaked dwarf virus (SRBSDV). Bull. Inst. Trop. Agric. Kyushu Univ..

[7] Jiang Y.Y., Guo R., Liu Y., Feng X. (2010). The occurrence and prevention profile of rice virus disease in Vietnam. Chin. Plant Prot..

[8] Wang Z.C., Yu D.D., Li X.Y., Zeng M.J., Chen Z., Bi L., Liu J.J., Jin L.H., Hu D.Y., Yang S. (2012). The development and application of a Dot-ELISA assay for diagnosis of southern rice black-streaked dwarf disease in the field. Viruses.

[9] Wang Z.C., Li X.Y., Wang W.L., Zhang W.Y., Yu L., Hu D.Y., Song B.A. (2015). Interaction research on the antiviral molecule Dufulin targeting on southern rice black streaked dwarf virus P9–1 nonstructural protein. Viruses.

[10] Bentur J.S., Viraktamath B.C. (2008). Rice planthoppers strike back. Curr. Sci..

[11] Chen Z., Guo R., Zhong L., Qiu G.H., Chen M.H., Song B.A., Liu J.J., Fan H.T., Li X.Y., Yang S. (2010). Cause of outbreak of southern rice black-streaked dwarf virus disease at Matian Township of Luxi County, Jiangxi Province. Guizhou Agric. Sci..

[12] Yan J., Kudo H., Uyeda I., Lee S.Y., Shikata E. (1992). Conserved terminal sequences of rice ragged stunt virus genomic RNA. J. Gen. Virol..

[13] Wu J.G., Du Z.G., Wang C.Z., Cai L.J., Hu M.Q., Lin Q.Y., Wu Z.J., Li Y., Xie L.H. (2010). Identification of Pns6, a putative movement protein of RRSV, as a silencing suppressor. Virol. J..

[14] Ling K.C., Tiongco E.R., Aguiero V.M. (1978). Rice ragged stunt, a new virus disease. Plant Dis. Rep..

[15] Ling K.C., Tiongco E.R., Aguiero V.M. (1978). Host range of rice ragged stunt virus. Int. Rice Res. Newslett..

[16] Ling K.C., Tiongco E.R., Aguiero V.M. (1977). Transmission of rice ragged stunt disease. Int. Rice Res. Newslett..

[17] Thanh D.N., Séverine L., Martine B., Hoang A.T., Do N.V., Pascal G., Christophe B. (2015). P2 of Rice grassy stunt virus (RGSV) and p6 and p9 of Rice ragged stunt virus (RRSV) isolates from Vietnam exert suppressor activity on the RNA silencing pathway. Virus Genes..

[18] Hoang A.T., Zhang H.M., Chen J.P., Hébrard E., Zhou G.H., Vinh V.N., Cheng J.A. (2011). Identification, characterization, and distribution of Southern rice black-streaked dwarf virus in Vietnam. Plant Dis..

[19] Xu H.X., He X.C., Zheng X.S., Yang Y.J., Tian J.C., Lu Z.X. (2014). Southern rice black-streaked dwarf virus (SRBSDV) directly affects the feeding and reproduction behavior of its vector, *Sogatella furcifera* (Horváth) (Hemiptera: Delphacidae). J. Virol..

[20] Huang H.J., Bao Y.Y., Lao S.H., Huang X.H., Ye Y.Z., Wu J.X., Xu H.J., Zhou X.P., Zhang C.X. (2015). Rice ragged stunt virus-induced apoptosis affects virus transmission from its insect vector, the brown planthopper to the rice plant. Sci. Rep..

[21] Cheng Z.B., Li S., Gao R.Z., Sun F., Liu W.C., Zhou G.H., Wu J.X., Zhou X.P., Zhou Y.J. (2013). Distribution and genetic diversity of southern rice black-streaked dwarf virus in China. J. Virol..

[22] Velusamy R., Heinrichs E.A. (1986). Electronic monitoring of feeding behavior of *Nilaparvatalugens* (Homoptera: Delphacidae) on resistant and susceptible rice cultivars. Environ. Entomol..

[23] Khush G.S., Brar D.S. (1991). Genetics of resistance to insects in crop plants. Adv. Agron..

[24] Keiichiro M., Tomomi T., Kazuhiro Y., Junichi S., Misturu O., Masatoshi O., Masaya M. (2015). Quantitative analysis of southern rice black-streaked dwarf virus in *Sogatella furcifera* and virus threshold for transmission. Phytopathology.

[25] Chen Z., Song B.A. (2011). The Prevention and Control Technology of Southern Rice Black-Streaked Dwarf Virus Disease.

[26] Wang F., Qin G.Z., Sui Z.H., Wang Z.H., Wang Z.Y., Yu J.L., Zhang J.R. (2006). Improved method for assaying maize plant resistance to maize rough dwarf disease by artificial inoculation and real-time RT-PCR. Eur. J. Plant Pathol..

[27] He P., Liu J.J., He M., Wang Z.C., Chen Z., Guo R., Correll J.C., Yang S., Song B.A. (2013). Quantitative detection of relative expression levels of the whole genome of Southern rice black-streaked dwarf virus and its replication in different hosts. Virol. J..

[28] Hu W.J., Chen J., Liu T.W., Simon M., Wang W.H., Chen J., Wu F.H., Liu X., Shen Z.J., Zheng H.L. (2014). Comparative proteomic analysis of differential responses of *Pinus massoniana* and *Taxus wallichiana* var. *mairei* to simulated acid rain. Int. J. Mol. Sci..

[29] Fang W.P., Xie D.Y., Zhu H.Q., Li W., Xu Z.Z., Yang L.R., Li Z.F., Sun L., Wang J.X., Nie L.H. (2015). Comparative proteomic analysis of *Gossypium thurberi* in response to *Verticillium dahliae* inoculation. Int. J. Mol. Sci..

[30] Yan S.P., Zhang Q.Y., Tang Z.C., Su W.A., Sun W.N. (2006). Comparative proteomic analysis provides new insights into chilling stress responses in rice. Mol. Cell. Proteom..

[31] Xia Y., Hong H., Ye L.C., Wang Y.L., Chen H.W., Liu J.F. (2013). Label-free quantitative proteomic analysis of right ventricular remodeling in infant Tetralogy of Fallot patients. J. Proteom..

[32] Chen Z., Guo Q., Chen B.H., Li X.Y., Wang Z.C., He P., Yan F., Hu D.Y., Yang S. (2013). Development of proteomic technology of shotgun and label free combined with multiple reaction monitoring to simultaneously detect southern rice black-streaked dwarf virus and rice ragged stunt virus. Virus Dis..

[33] Cox J., Neuhauser N., Michalski A., Scheltema R.A., Olsen J.V., Mann M. (2011). Andromeda: A peptide search engine integrated into the MaxQuant environment. J. Proteome Res..

[34] Luber C.A., Cox J., Lauterbach H., Fancke B., Selbach M., Tschopp J., Akira S., Wiegand M., Hochrein H., O’Keeffe M. (2010). Quantitative proteomics reveals subset-specific viral recognition in dendritic cells. Immunity.

[35] Sta C.F.C., Hull R., Azzam O. (2003). Changes in level of virus accumulation and incidence of infection are critical in the characterization of *Rice tungro bacilliform virus* (RTBV) resistance in rice. Arch. Virol..

[36] Fang X.P., Chen J.P., Dai L.Y., Ma H.S., Zhang H.M., Yang J., Wang F., Yan C.Q. (2015). Proteomic dissection of plant responses to various pathogens. Proteomics.

[37] Ryals J., Uknes S., Ward E. (1994). Systemic acquired resistance. Plant Physiol..

[38] Luna E., Bruce T.J.A., Roberts M.R., Flors V., Ton J. (2012). Next-Generation Systemic Acquired Resistance. Plant Physiol..

[39] Durrant W.E., Dong X. (2004). Systemic Acquired Resistance. Annu. Rev. Phytopathol..

[40] Maldonado A.M., Doerner P., Dixon R.A., Lamb C.J., Cameron R.K. (2002). A putative lipid transfer protein involved in systemic resistance signalling in Arabidopsis. Nature.

[41] Zhang Q., Sun T.J., Zhang Y.L. (2015). ER quality control components UGGT and STT3a are required for activation of defense responses in *Bir1–1*. PLoS ONE.

[42] Noutoshi Y., Kuromori T., Wada T., Hirayama T., Kamiya A., Imura Y., Yasuda M., Nakashita H., Shirasu K., Shinozaki K. (2006). Loss of necrotic spotted lesions 1 associates with cell death and defense responses in Arabidopsis thaliana. Plant Mol. Biol..

[43] Morita-Yamamuro C., Tsutsui T., Sato M., Yoshioka H., Tamaoki M., Ogawa D., Matsuura H., Yoshihara T., Ikeda A., Uyeda I. (2005). The Arabidopsis gene CAD1 controls programmed cell death in the plant immune system and encodes a protein containing a MACPF domain. Plant Cell Physiol..

[44] Elvira M.I., Galdeano M.M., Gilardi P., Garcia L., Serra M.T. (2008). Proteomic analysis of pathogenesisrelated proteins (PRs) induced by compatible and incompatible interactions of pepper mild mottle virus (PMMoV) in *Capsicum chinense* L3 plants. J. Exp. Bot..

[45] Zhou X.J., Lu S., Xu Y.H., Wang J.W., Chen X.Y. (2002). A cotton cDNA (GaPR-10) encoding a pathogenesis-related 10 protein with in vivo ribonuclease activity. Plant Sci..

