# Supplementary material for: Evaluation of Rice Resistance to Southern Rice Black-Streaked Dwarf Virus and Rice Ragged Stunt Virus through Combined Field Tests, Quantitative Real-Time PCR, and Proteome Analysis
Source: Viruses. 2017 Feb 22;9(2):37. doi: 10.3390/v9020037 (PMC5332956; doi:10.3390/v9020037)
Supplement: Supplementary file 1 [file viruses-09-00037-s001.zip › viruses-164039-supplementary-Figure S1 Figure S2 Figure S3.doc]

Supplementary Materials: Evaluation of Rice Resistance to Southern Rice Black-streaked Dwarf Virus and Rice Ragged Stunt Virus through Combined Field Tests, Quantitative Real-time PCR, and Proteome Analysis

**Figure S1.** Detection for specificity of primers designed for SRBSDV by conventional PCR. Lanes 1 to 13 represent the amplified products of partial SRBSDV genes (S1, S2, S3, S4, S5-1, S5-2, S6, S7-1, S7-2, S8, S9-1, S9-2, and S10). Lane 14 represents 18S rRNA. M: M, DL 500 bp DNA marker.

**Figure S2.** Detection for specificity of primers designed for RRSV by conventional PCR. Lanes 1 to 12 represent the amplified products of partial RRSV genes (S1, S2, S3, S4-1, S4-2, S5, S6, S7, S8-1, S8-2, S9, and S10). M: M, DL 500 bp DNA marker.

**Figure S3.** Hierarchical clustering analysis of differentially expressed proteins in Z1, L2186, and FYXZ. Eighteen columns represent healthy and SRBSDV stress treatments of the three rice varieties. Three repetitions of A, B, C, D, E, and F, represented SRBSDV infected FYXZ (WFYXZ), healthy FYXZ (HFYXZ), SRBSDV infected L2186 (WL2186), healthy L2186 (H L2186), SRBSDV infected Z1 (WZ1), and healthy Z1 (HZ1), respectively. The rows represent individual proteins. The up- and down-regulated proteins are indicated in red and green, respectively. The intensity of the colors increases as the expression differences increase, as shown in the bar on the right.
